# Processes and challenges of community mobilisation for latrine promotion under *Nirmal Bharat Abhiyan* in rural Odisha, India

**DOI:** 10.1186/s12889-017-4382-9

**Published:** 2017-05-16

**Authors:** Parimita Routray, Belen Torondel, Marion W. Jenkins, Thomas Clasen, Wolf-Peter Schmidt

**Affiliations:** 1 Faculty of Infectious and Tropical Diseases, London School of Hygiene and Tropical Medicine, London, UK; 2 Department of Civil and Environmental Engineering, University of California Davis, One Shields Ave, Davis, CA 95616 USA; 3 Department of Environmental Health, Rollins School of Public Health, Emory University, Atlanta, GA USA

**Keywords:** Nirmal Bharat Abhiyan, Community mobilisation, Sanitation promotion, Caste and power dynamics

## Abstract

**Background:**

Despite efforts to eradicate it, open defecation remains widely practiced in India, especially in rural areas. Between 2013 and 2014, 50 villages in one district of Odisha, India, received a sanitation programme under the *Nirmal Bharat Abhiyan* (NBA – “Clean India Campaign”)*,* the successor of India’s Total Sanitation Campaign. This paper documents the strategies and processes of NBA community mobilisation for latrine promotion in these villages and assesses the strengths and limitations of the mobilisation activities.

**Methods:**

NBA’s community mobilisation activities were observed and assessed against the programme’s theory of change in 10 randomly selected programme villages from start to finish. Additional data was collected through review of documents, individual interviews (*n* = 80) and focus group discussions (*n* = 26) with staff of the implementing NGOs and community members.

**Results:**

Our study revealed the lack of a consistent implementation strategy, lack of capacities and facilitation skills of NGO staff to implement sanitation programmes, political interference, challenges in accessing government financial incentives for latrine construction, and lack of clarity on the roles and responsibilities among government and NGO staff, leading to failure in translating government policies into sustainable actions. Social divisions and village dynamics related to gender and caste further constrained the effectiveness of mobilisation activities. Meetings were often dominated by male members of upper caste households, and excluded low caste community members and views of women. Community discussions revolved largely around the government’s cash incentive for latrines. Activities aimed at creating demand for sanitation and use of latrines often resonated poorly with community members. An assessment by the implementers, 1 year after community mobilisation found 19% of households had a completed latrine across the 50 villages, a marginal increase of 7 percentage points over baseline.

**Conclusions:**

In this setting, the Government of India’s NBA programme to increase rural sanitation coverage and use is hampered by political, programmatic, logistical and socio-structural constraints. Sanitation demand generation was difficult for local implementing NGOs as village populations had lost trust in organisations due to previous indications of fraud. Agencies or organisations implementing sanitation campaigns and conducting sanitation promotions need to enhance their staff’s knowledge and build capacity in order to address important social heterogeneity within villages. This trial’s registration number is NCT01214785 (October 4, 2010).

## Background

Widespread open defecation remains a major cause of transmission of diarrhea [1, 2], worm infection and trachoma worldwide [3]. More than half of the world’s population who defecates in the open, resides in India [4]. In the 2011 Indian census, 67% of rural and 13% of urban households reported defecating in the open [5]. Much of the disease burden associated with diarrhea and stunting in India are thought due to lack of sanitation [6]; and improving sanitation could significantly reduce this heavy burden [7, 8].

The Indian government’s attention to sanitation and nationwide interventions promoting rural sanitation started in the 1980’s. The Central Rural Sanitation Programme (CRSP), the first large-scale initiative, was launched in 1986. Households were given subsidized hardware to build latrines. The CRSP failed to achieve and sustain high levels of sanitation coverage and usage [9]. In 1999, CRSP was restructured as the Total Sanitation Campaign (TSC) with an aim to end open defecation [10]. To encourage communities to reach full sanitation coverage, Clean Village Awards (*Nirmal Gram Puraskar*) were introduced in 2003 to offer cash prizes to villages that achieved open defecation free status [11, 12]. In a revised guideline in 2007 [11], TSC adopted a ‘community-led’ and ‘people-centered’ strategy in which intensive IEC (information, education and communication) activities would lead to increased awareness, changes in open defecation social norms and behavior, and demand for sanitary facilities among rural people.

As in the CRSP, the Government of India (GoI) and state governments continued to extend financial subsidies to individual households to build latrines under TSC, but restricted the support to households below the poverty line (BPL) and changed it from an input subsidy to an output ‘incentive’, by revising the delivery mechanism from a hardware input under CRSP to a post-construction cash payment [10]. These efforts increased latrine coverage, but many households that built latrines continued to defecate in the open despite owning a functional latrine at home [9, 11–16]. Evaluations of many TSC programmes found that the primary reasons why many failed to generate the expected large gains in rural demand for and use of latrines, were an over-emphasis on latrine construction and ineffective implementation of behavior change processes and IEC activities [11–13, 15].

In 2012, the GoI initiated *Nirmal Bharat Abhiyan* (NBA, “Clean India Campaign”) to succeed the TSC with a goal to achieve 100% sanitation access to all rural households by 2022 [17]. This new campaign aimed to accelerate rural sanitation coverage by continuing the ‘community-led’ and ‘people-centred’ strategies of the TSC, with increased emphasis on community mobilisation, collective and sustainable behaviour change, and IEC activities. Financial incentives support continued for building latrines and were offered to more households, both BPL and those identified as above poverty line (IAPL). In October 2014, the NBA was relaunched as (SBA, “Clean India Mission”) to achieve *Swachh Bharat* (“Clean India”) by 2019 [18]. The SBA approach is also ‘community-led’ with the addition of ‘community saturation’, putting a central focus on awareness raising and triggering collective behaviour change [18].

In each of these recent sanitation campaigns (TSC, NBA and SBA), the promotion approach has remained very similar but the financial incentive for building individual household latrine climbed from 3200 Indian rupees (about US$48) at the end of TSC, to minimum 4600 to 9100 rupees (US$69 -US$ 136) maximum in NBA, to 12,000 rupees (US$179) in SBA [10, 17, 18]. However, the funding for IEC and mobilisation activities in TSC and NBA remained unchanged at 15% of the total project costs [10, 17].

Community mobilisation is a participatory communications approach that seeks to engage the whole community as individuals and as groups, including marginalised populations, to identify their problems, suggest solutions and initiate actions themselves [19]. Participatory methodologies to promote community-wide hygiene, sanitation, and community management of water and hygiene facilities, were formally first developed in the 1980’s under the PHAST (Participatory Hygiene and Sanitation Transformation) community-led approach and implemented in African countries and elsewhere during the 1990s [20]. PHAST was an adaptation of SARAR (Self-esteem, Associative strengths, Resourcefulness, Action planning and Responsibility) from the 1970s to motivate communities to improve sanitation and hygiene [21].

Participatory approaches at community level have proved to be effective in changing sanitation behaviours and encouraging latrine adoption in rural Bangladesh, India, Zimbabwe, Ethiopia, and elsewhere. Community-Led Total Sanitation (CLTS) is one example of a participatory approach developed in Bangladesh in the year 2000, to change sanitation behaviours in communities specifically to end open defecation, whose success led to its adoption in communities of Asia and Africa [22, 23]. CLTS adapted PHAST tools and participatory rural appraisal (PRA) techniques and features like ‘disgust’ and ‘shame’, to trigger community members to realise that open defecation causes them to ingest each other’s faeces and creates risks to the health of the whole community, and to take action to totally end it. Community health clubs, another community-led participatory methodology originally from Zimbabwe was designed to develop community cohesion and a culture of health among the target population, which subsequently led to large increases in latrine coverage, and its success led to its replication in Asia and Africa [24]. Similarly, a community mobilisation programme for sanitation developed locally in Amahara District, Ethiopia in 2004 resulted in large increases in basic latrine ownership within a year, without any financial incentives or subsidies for construction [25].

Effective community mobilisation interventions for sanitation promotion in India that used various participatory approaches including CLTS also had success increasing the latrine coverage and stimulating adoption and use. Community mobilisation intervention in Neen Gram Panchayat in Shimla District, Himachal Pradesh, led to ODF status in 3 months and a similar mobilisation, radically transformed the traditional lifestyle of a tribal community in Koraput District, Odisha, to become ODF [26]. An intensified IEC campaign and social mobilisation for latrine construction within the framework of TSC in Odisha’s Bhadrak District also had a substantial effect on latrine adoption and use [27].

This paper presents the processes and challenges of conducting community mobilisation for latrine promotion under NBA. The key objectives of this study were to: 1) assess the strategies and processes of community mobilisation and community triggering for latrine demand generation as implemented under the NBA, and 2) examine the challenges of executing community-based mobilisation for sanitation promotion in rural communities comprising diverse people of different castes.

## Methods

### Study setting

This study was undertaken as part of a cluster randomized health impact trial of the TSC intervention in 100 villages of Puri District, Odisha, India [28], where the 50 intervention villages received the TSC programme between 2011 and 2012. The trial’s details and the latrine promotion under TSC are described elsewhere [28, 29]. Following the conclusion of the trial, the 50 control villages received the NBA sanitation intervention between 2013 and 2014, as the TSC had been replaced by NBA.

**Fig. 1:**
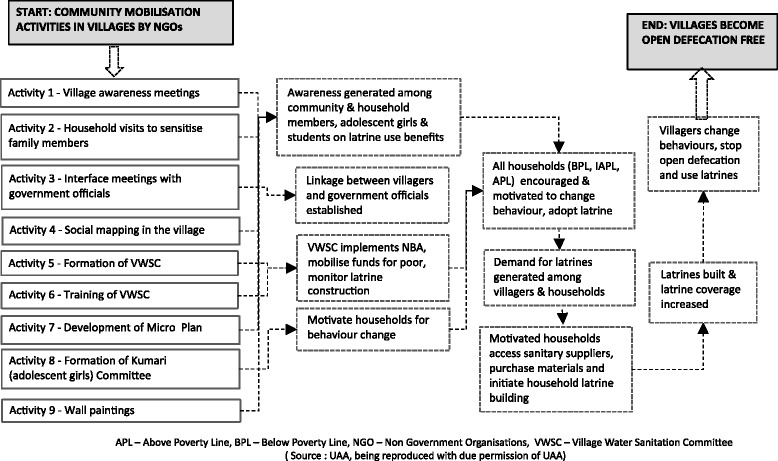
Theory of change: Community mobilisation results in raising demand, acquisition, and use of latrines among rural households of Puri District

This study examines the roll out of the NBA’s community mobilisation for latrine promotion in the 50 control villages, and compares them to the programme’s theory of change and to the TSC community mobilisation (described elsewhere) as implemented in the 50 intervention villages [29]. The theory of change shown in Fig. 1, was developed jointly by the lead agency and the intermediary organisation, that involved a series of mobilisation activities, leading to the expected changes and outcomes like higher motivations and demand for sanitation among villagers, change in their sanitation behaviours and end of open defecation. This study was approved by the ethics committees of the London School of Hygiene and Tropical Medicine and the local collaborator - Xavier University, Bhubaneswar, Odisha.

### NBA’s community-led approach

An intensive IEC campaign was the corner stone of NBA. Each Indian state was to develop its own IEC strategy, including methods such as folk, mass and outdoor media like wall painting and hoarding. Each district then prepares a detailed IEC plan using additional strategies as needed engaging all sections of the community including the *Panchayati Raj* Institutions (PRI - local government), cooperatives, school teachers, community health workers, *Anganwadi* (pre-school nursery centre) workers, women groups, self-help groups (SHGs), etc. To strengthen communication within villages, motivators (*Swachchhata Doot -* “Sanitation Messenger”) were to be engaged [17]. Local NGOs of repute could be contracted to implement community mobilisation activities, conduct interpersonal communication (IPC) activities, select motivators, execute IEC such as wall paintings and street plays, and organise capacity building and training of village water and sanitation committees (VWSC), PRIs and grass root functionaries.

### NBA’s financial incentive for latrine construction

The NBA guidelines prescribed a completed household sanitary latrine to be a ‘Latrine Unit’ including a super structure constructed by the ‘household itself’, and upon completion and verification by government officials, the cash incentive was to be directly transferred to the household’s bank account. The financial incentive for a completed latrine was 4600 rupees (US$ 70). All BPL and IAPL households belonging to Scheduled Caste (SC - lowest caste, considered ‘untouchable’) or Scheduled Tribe (ST - socially disadvantaged indigenous people) were eligible for the financial incentive. Other APL households were supposed to self-finance and construct their own latrines. To further help the BPL households with additional construction costs, the government’s Mahatma Gandhi National Rural Employment Guarantee Scheme (MGNREGS) was aligned with NBA, that allowed BPL households reimburse from MGNREGS for up to 26 days of labour towards constructing their latrine, a cash value of 4500 rupees (US$ 69.2) resulting in up to 9100 rupees (US$ 140) under the NBA-MGNREGS agreement.

### Data collection and analyses

For each implementing NGO, two assigned villages were randomly selected for a total of 10 study villages. The research team had access to implementer’s field plan prior to the start of mobilisation activities, so the team attended the NGO’s staff training and the community-based mobilisation and promotional activities (shown in Fig. 1) in each study village, to observe the settings and the context in which they were delivered, assess and compare them to TSC implemented villages. Two researchers who could understand, read, write and speak *Odia* (the local language) attended, observed and documented the processes of these promotions. Data collection began in August 2013 with the start of the training of NGO staff and continued until April 2014. Attending the training helped to understand the implementer’s strategy/plans for community mobilisation and guided development of data collection tools. Table 1 outlines the research questions, methods, indicators and tools used in this study.

**Table 1:** Sanitation promotion under NBA: Assessment objectives, indicators and data collection tool

Aspects of Community Mobilisation	Data collection method	Indicators	Data Source
Aim 1:To assess the strategies and processes of community mobilisation and community triggering for rural sanitation demand generation under the NBA			
NGO field staff training	Document review, Attending the trainings/ Direct observation, FGDs with facilitators (*n* = 6)	Enhanced knowledge of facilitators	Training manual and presentations; Researcher’s observations; FGD notes
Staff’s action plans of implementing NGOs	Documents review	Existence of NGO’s field action plans	Field/Action plans
Facilitators’ understanding of the intervention design/plan	FGDs with facilitators	Existence of activity plans of field staff	NGO’s field plans; FDG notes
Formation of different committees	Document review, Attending the meetings	Committee members executing the campaign	Committee registers
Community's understanding of the intervention	FGDs with villagers (*n* = 20)	Villagers’ awareness on objective of NBA; Changed perception and attitude towards OD; Demand for latrines generated	FGD notes
Aim 2: Examine the challenges of executing NBA in socially diverse villages			
Challenges in organizing community mobilisation	Attending and observing village mobilisation activities	Community understands latrine’s importance and unites to implement NBA	Researcher observations FGD and IDI notes; Researchers observations
	FGDs with villagers (*n* = 20) IDIs with (*n* = 80)	Challenges identified by community members; Existence of IEC materials, their distribution among people and their effective use for awareness generation and behaviour change	
Facilitators’ understanding of the challenges	FGDs with facilitators	Challenges identified by NGO staff	FGD notes

The assessment involved qualitative data collection of the mobilisation activities (as in Fig. 1) by attending and observing; review of records and reports of NGOs and different committees at village level, conducting Focus group discussions (FGDs) with NGO field staff (*n* = 6) and community members (*n* = 20); and in-depth interviews (IDIs) with community members (*n* = 80) who had participated and witnessed promotional activities (Table 1).

In each study village, two FGDs were held with community members, one each with men and women (comprising 6 participants), 2 weeks after the NGO had completed all their mobilisation and sanitation promotions, so that participants could recall the NGO’s activities. FGD respondents were purposefully sampled for their insights and experiences into specific components or processes. FGDs lasted for 1–1.15 h and were facilitated by the lead researcher, who was fully conversant in *Odia*.

Similarly, 8 individuals (2 each of adult men and women, 2 each of adolescent [ages 16–18 years] girls and boys) per study village were interviewed about: 1) different aspects of community mobilisations by the NGO; 2) their understanding of the NBA; 3) how the mobilisation activities resulted in triggering people to demand for latrines; and 4) challenges posed by the communities to the NGOs during mobilisations. Using a sample frame from the attendees at the initial community meetings, we purposefully selected participants representing different community demography. The IDIs lasted for 25–30 min and to help ensure data quality, the lead investigator was present in half of the IDIs. FGDs and IDIs were audio recorded, transcribed and translated into English for analysis.

All data collection tools were developed in English and translated to *Odia*. The FGD and IDI protocols were piloted to check acceptability, feasibility and accuracy prior to use. All the qualitative data (transcripts of FGDs and IDIs and the observation notes) were collated and analysed using a thematic approach and the steps involved were: (1) familiarisation with the data by reviewing transcripts and notes and listing recurrent themes; (2) development of a coding framework for emerging themes; (3) coding the data and annotating the transcripts and observation notes; (4) rearranging the data according to the appropriate theme in N.Vivo (QSR International); and (5) interpreting the thematic data and identifying association between themes.

## Results

### Programme preparation process

#### Programme structure

The implementation of NBA in the 50 villages was led by WaterAid, an international water, sanitation and hygiene (WASH) NGO that contracted an Odisha based NGO - the United Artists' Association (UAA) with substantial experience in WASH, to facilitate the community mobilisation and latrine promotion. A Memorandum of Understanding (MOU) was signed between WaterAid (hereinafter referred as ‘lead agency’) and UAA (hereinafter referred as ‘intermediary organisation’) to facilitate NBA implementation in the villages. The village level mobilisations and promotions was subcontracted to 5 local NGOs (10 activities shown in Fig. 1), but UAA was responsible to coordinate the implementation between the local NGOs, the government representatives and the concerned departments and its officials. Specific criteria was not outlined for the selection of local NGOs, but all of them had 5 or more years of experience implementing WASH and had been past partners of the lead agency.

Each NGO formed a field team of four members - one Cluster Coordinator (CC) and three Gram Panchayat Sanitation Facilitators (GPSFs). Each GPSF was assigned 3–4 villages; their field plans were discussed and finalised in consultation with their CC. CCs were the focal points responsible for overall implementation in the 11–12 villages and tasked with supporting the GPSFs in the delivery of all activities and monitor their work. The delegation of work and the staffing for the field execution were the same as that of the TSC villages [29].

#### Training of NGO field staff

In August 2013, with guidance and support of the lead agency, the intermediary organisation conducted a 3 days training for the NGO field team. Topics covered were (i) poverty and its relation with sanitation; (ii) millennium development goals and the country’s sanitation targets; (iii) faecal-oral transmission routes [30]; (iv) PRA techniques for mobilising people; (v) preparation of village’s social map; (vi) NBA’s features and guidelines; and (vii) lead agency’s sanitation promotion strategy and their achievements. These topics were largely the same as those covered in the NGO field staff training for TSC villages [29]. Power point presentations, games, group exercises and picture cards (with drawings of sanitation and hygiene scenarios) were the methodologies used in the training.

While being occasionally supported by experts from private institutes and District Water and Sanitation Mission (DWSM - a district level government agency that promotes WASH), sessions were led by just one male staff of the intermediary organisation, who seemed to lack adequate knowledge on some of the mentioned training topics. DWSM experts were unable to remain present for the full sessions allotted to them, to explain the NBA guidelines and government’s strategy for implementation. Therefore, many of the questions of staff regarding components of NBA were left unheard, unanswered and not clarified. Staff’s roles and responsibilities were not explained in the trainings, but latrine construction and achieving latrine targets in a certain timeframe was emphasised throughout the training, though latrine construction not being NGOs’ responsibility under the NBA (see quotes Table 2, Lack of clarity among NGO staff).

**Table 2:** Study participant’s quotes on sanitation promotion and community mobilisation activities

Topic	Detailed quotes
Poor quality of training of NGO field staff	• *Training was not enough, and we should have had another training programme. We still have doubts as to how it [NBA] will be done, who will do it and when will it happen. (FGD – NGO)* • *When people say we don’t have sufficient funds to build a latrine, then, we don’t know how to motivate them. (FGD – NGO)* • *In a community there are different ways of educating them but we were not given those aids and tools. (FGD – NGO).* • *At the time of recruitment, we perceived funds to be given to us by the intermediary NGO or government to build latrines in the villages. It is only in the training in Puri, we got to know that through ‘awareness generation and mobilisation’ only, we have to facilitate the latrine building in villages and achieve the latrine targets, which is difficult. (FDG – NGO) * • *At the end of the training, we were given latrine targets to be built each month, although we were not directly responsible for latrine construction. (FGD – NGO)*
Lack of clarity among NGO staff	• *When we visit the villages for mobilisation meetings, whatever strikes to my mind, I tell them. If I have experience on HIV, then, I tell them about HIV and other health issues even if it is not related to sanitation. Whatever little I know about sanitation, I just tell that to those villagers. (FGD – NGO)* • *We have learnt only 7–8 points from the 3 days training and when it comes to applying these learnings in the field to motivate people, these 7–8 points is insufficient. Then, we apply our existing knowledgebase and not the knowledge gained from the trainings.(FGD – NGO)* • *Whatever we have in our brain, we speak and discuss those, as much as possible and when our stock get exhausted, then we keep mum. (FGD –NGO)* • *I have joined now and villages are new for me, so I wanted more training and clarity on mobilizing government funds, but old staff said they don’t need any training. I was then told to concentrate on construction and let go the training*. (*FGD - NGO)*
Programme initiation: Political interference	• *There are interferences by political parties and the local politicians. The local leaders dictate us [NGOs] in which village to work/ not work. And if we don’t listen to them, then they do not let us to enter the village or allow holding any meetings. They also directed in which hamlets, the latrines are to be built/ left out. They even threatened to take our lives, if we did not listen to them. (FDG – NGO)*
Programme initiation: value of door-to-door-visits	• *The NGO man visited our houses and explained about the latrine programme to all the members present in the house and answered our queries too. His visiting our house was nice, and we could learn more about the latrine building work [programme] of government. By this, the female family members who would not have participated in the village meetings and in the midst of the crowd, could participate and interact with them. (IDI – adult female)*
Logistical constraints in mobilising people for community meetings	• *Gathering people at a common place is very time consuming. There are people who do not come to meetings, even if they are informed in advance. Some come as per their wish. So, we have to wait and sit [convene the meeting] according to villager’s availability and time. Whichever time they gave us, we made ourselves available in the village, which could be a Sunday or Saturday, and sometimes in the evening. (FDG – NGO)* • *Ours is a big village, and it is difficult to bring people of all castes and sections to one location and do the meeting. (IDI- adult female, general caste)*
Village awareness meeting: challenges engaging women	• *In many villages, we held meetings in a public place like schools instead of a temple [as some lower caste people are not allowed to enter the temple premises] so that all sections of the village, men and women could attend. In the meetings, women always sat at the back, behind the men and mostly do not voice their opinions, which was a challenge to engage women in the discussions and to get their views and feedback. It’s because our society does not allow them to sit in the front. (FDG - NGO)* • *There are gender related issues, the challenge of bringing people of both sex to the same platform. To mobilize men and women and getting them to sit in one platform was difficult. Men and women do not agree to sit together [share the same space]. Married women said they will not sit along with men, as they may touch their (elder) brother-in-laws [referring to the social norm]. Despite trying several times, we failed to bring them together. (FGD – NGO)* • *Due to this social norm of women reluctant to share the same platform with men, we had to hold separate meetings with women and men folks. Due to this, we also had challenges in formation of Village Water Sanitation Committee. Some members for the committee were nominated by men and the rest by women, in separate groups. (FGD – NGO)*
Village-wide meetings: exclusion of low caste members	• *While conducting a meeting at the mandap [a raised platform next to the temple], general caste people are unwilling to let the lower /scheduled caste (SC) people sit with them and were made to sit on the floor. During a VWSC formation, SC people had to sit away from the general caste people. (FGD –NGO and Observation)* • *We heard of a meeting taking place in the main village to discuss latrine construction but, we were not called to the meeting. Only 2–3 elders from our hamlet attended it. (IDI – adult female, low caste hamlet)* • *We are low caste people, and higher caste people did not like our joining the meetings. (IDI – adult male, low caste).* • *What’s the point in attending such [interface] meetings where all the decisions are done by high class and influential people and our views are not even heard.(IDI – Adult male)*
Village awareness meeting: fixation with construction subsidies	• *Don’t we know about open defecation, and impact? Don’t beat around the bush. Who has time to listen to all these things? Come to the main topic. Just tell how much you have got to pay us for the latrine*. *(Observation– village mobilisation meeting)* • *The NGO had a meeting, and told about dignity loss and shame by defecating in the open. This is nothing new, we already know, it is also shown on TV. Instead, if the NGO have got funds for latrines, then, they should immediately start building them. (IDI – Adult Male)* • *We were told that the NGO has brought the latrine programme to our village and if we do not attend the NGO meeting, we will be excluded from the latrine construction list. Therefore, I have come to attend this mapping meeting*. (*IDI – Adult male)*
VWSC membership: exclusion of low caste	• *Why nominate those SC people for the committee. They are illiterate and it will not be useful to have them in the committee*. *(Observation – village mobilisation meeting)*
*Kumari* Committee: perceived value	• *Adolescent girls are a good channel/ medium to influence the parents on sanitary habits which would later facilitate latrine adoption. (FGD – NGO)* • *If a son asks his father for something, the father may not listen or turn it down, whereas if the daughter demands it, then the father listens and pays attention. (FGD - NGO)* • *If a daughter tells her father that I am now grown up and feel shy to defecate in the open, the father would construct a latrine for her. Whereas, if a son repeatedly asks the father, he would not do it. (FGD - NGO)*
*Kumari* Committee: lack of purpose	• *We were told to clean the village by the NGO, which all girls did a couple of times with other villager’s help. But after those few cleaning events, everything stopped. Now we do not do anything as no one directed us, what we should be doing next. So we don’t know what we are supposed to do as a member of the Kumari Committee and what our deliverables are?(IDI –Kumari Committee member)*
Social mapping: used to track open defecation	• *Through maps, we show the villagers the vulnerable or contamination points caused by open defecation, and after latrines are constructed and used, they can see the transformation of these contamination points to better places. (FDG- NGO).*
Wall painting: dissonant NGO and villager perceptions	• *By seeing the wall painting, they [villagers] identified the open defecation sites and also felt guilty for their acts and realised what their village’s sanitation situation was. (FDG – NGO)* • *These NGOs have received funds from government to build latrines for our village, but they have siphoned off money meant for us, and in return only doing this painting. (IDI – adult male)*

Newly joined staff and those lacking prior experience implementing sanitation campaigns or WASH, found the trainings ‘not adequate’. Some experienced staff were dissatisfied and felt these trainings as general orientation to different sanitation related topics, which did not help enhance their capacities, acquire new knowledge or skills essential to take up the bigger role of mobilising and facilitating participatory approaches for behaviour change and sanitation uptake among villagers (see quotes Table 2, Poor quality of training for NGO field staff). Apart from this one training, no further refresher training over the 9-month mobilisation period were organized, despite staff’s request for more trainings on aspects like PRA, etc. The field staff largely applied their existing knowledge, which could be unrelated to sanitation.

### Process of community mobilisation

#### Introductory meetings and programme initiation

GPSFs and CCs visited each village and mostly met the *Sarpanch* (elected local Panchayat head) or the village ward member to inform them of their visit purpose, and fix a date for conducting an initial awareness meeting for all villagers. They delegated a responsible and respected influential villager to call a meeting for the said date. In this first or in subsequent visits, they also got introduced to others - such as the Panchayat *Samiti* (committee) member, *Anganwadi* worker, adolescent girls and women SHG members. However, in two villages of two different NGOs, the introductory meetings could not be held due to resistance by some local politicians (for reasons related to corruption explained in discussion). All efforts by NGO staff, the lead agency and the intermediary organisation were blocked and none were able to rectify the situation. As a result, these two villages were dropped from the NBA intervention. Similarly, field staff in a few other villages were frequently pressured by local politicians to prioritise or de-prioritise certain communities (see quotes Table 2, Programme initiation: political interference).

One of the 5 NGOs adopted a different approach. They made door-to-door visits where households were informed about NBA’s implementation in their village and this gave the staff an opportunity to meet all family members and sensitise them about sanitation and latrine’s importance. The value of these home visits and how more effective they were for engaging women in the process compared to the general village awareness meetings, is illustrated by one villager’s quote (see Table 2, Programme initiation: value of door-to-door visits). From a gender inclusive community participation perspective, door-to-door visits appeared to be a promising way to increase awareness and demand for sanitation, especially among females.

#### Village awareness meetings

Awareness meetings gathering villagers at a public place was convened by NGO staff in each village, where they mostly delivered a speech touching on topics around health impacts and dignity loss especially of women by open defecating, and the importance of latrines. Latrine construction was greatly emphasized and no other triggering activities were done. Patriarchal logic were used to promote the construction of latrines and the messages frequently conveyed were:

- *Defecating in the open is shameful especially for women.* (*NGO staff*)
- *We should feel ashamed that women are seen with their private parts exposed while defecating in fields, whereas at home, they are asked to keep their heads covered, under veil.* (*NGO staff*)

We observed, villagers were often clearly uninterested in these topics but higher financial incentive of NBA lured many villagers to attend and they were impatient to learn about the budget the NGO had for latrines, and how the latrine would be built (see quotes Table 2, Village awareness meeting: fixation with construction subsidies). Staff spent considerable time explaining the funds available for latrines under NBA and MGNREGS and ways to access them.

Motivated by the cash incentive, many households initiated latrine construction preparations just a few days after these meetings, expecting the NGO to build latrines for them, advance all costs and organize transportation of construction materials (as practiced under the TSC), and themselves making only the nominal cash and labour contribution. Upon this not happening, many who lacked funds to finance construction on their own, abandoned their efforts. Many refused to resume the construction, if they did not get paid the initial money spent on construction. The change in supply-side execution modalities in NBA, in which NGOs were no longer pre-financing and managing the latrine construction on behalf of eligible households, made villagers realize that they were no longer dependent on the NGOs to get the cash incentive reimbursed from the government. So they did not consider these awareness meetings important. This posed challenge for the NGOs to mobilize more people to these meetings and motivate them for latrine adoption.

An important objective of these awareness meetings was to regain lost trust during previous sanitation campaigns, in which some other NGOs and individuals had fraudulently collected money from households, with the promise to build latrines. NGOs used this forum to issue alerts to villagers to not give money to anyone coming in the name of latrine construction. Many villagers did not attend these meetings, when they learnt they were ineligible to receive any cash incentive, as they already claimed one in government’s previous sanitation programmes. With low level of participation in these meetings, it became difficult for the field staff to prepare a comprehensive list of households eligible for the cash incentives.

In one out of 10 awareness meetings, we observed cards containing pictures of sanitation and hygiene scenarios being used to enable villagers distinguish between good and bad sanitation and hygiene practices. This picture card set was developed by the lead agency for a past sanitation programme and had not been pre-tested for use in NBA’s mobilisation activities, but each NGO was provided with only ‘one’ set of these cards. Without a card set for each staff to carry to the field, NGOs stored the set in their offices. Other than these meetings, staff mentioned using these cards during household visits and group meetings. In IDIs with villagers, none mentioned being shown any picture cards. Other than this card set, staff were not equipped sufficiently with audio - visual aids, essential to be used as channels to sensitise, and generate interest among villagers for latrine adoption, though it was the intermediary organization’s responsibility to make these resources available to the NGOs staff. With this not happening, leaflets with features of NBA (by DWSM for free distribution) were obtained by only two NGOs and were seen being distributed in only two meetings.

We observed several other challenges encountered by the staff in mobilizing people and convening awareness meetings such as fixing a meeting date and time that suited most villagers. Fundamental logistical constraints were faced in bringing together villagers of different castes to the meeting venue and facilitating the discussion especially engaging all in the discussion (see quotes Table 2, Logistical constraints in mobilizing people for awareness meetings). In most study village’s meetings, a small portion of the population (mostly adult and young men from higher castes) attended and voiced their opinion. In all cases, though women joined these meetings, their numbers were very low compared to men and refused to sit with men. They allowed the men to speak on their behalf and themselves did not participate in these discussions (see quotes Table 2, Village awareness meetings: challenges engaging women). There were no systematic attempts either by higher caste people or by the staff to overcome existing caste divisions and engaging lower caste people in these meetings and in the discussions (see quotes Table 2, Village wide meetings: exclusion of low caste members).

#### Interface meeting between NGO staff, villagers and local government representatives

Convened by NGO staff, with representation from lead agency and intermediate organisation, the interface meeting meant to introduce villagers to key government officials ((block level officer(s), engineer(s)) in charge of NBA’s and MGNREGS’s financial incentive processing, so that households could directly access their help for funds reimbursement. The ward member was present in most meetings, but the *Sarpanch* and the government officials, whose presence was considered important, were largely absent. This forum was eventually used to validate the village social map and prepare the list of eligible households for the cash incentive. Far fewer villagers attended interface meetings than attended awareness meetings. SC people (considered lower caste) were not allowed to share the same sitting space with that of higher castes. Similarly, women did not sit with men (for the same reasons as described earlier). In villages dominated by Brahmin (highest caste) households, the discrimination for lower caste people sharing the same platform space was particularly pronounced especially when interface meetings were held in temples, which occurred in 3 study villages. NGO staff were also not seen making dedicated efforts to mobilise more villagers to attend and those that attended could not differentiate between the objectives and outcomes of interface and awareness meetings.

#### Village water and sanitation committee (VWSC) formation and training

VWSC comprised of 15–20 members with representation from all sections, castes, and with equal numbers of men and women and it’s formation was the responsibility of the local implementing NGO. The role of VWSC as explained by NGOs was to facilitate sanitation implementation in villages, like identify space and take decisions about allocating communal land for poor landless families lacking land to build latrine, mobilise credit or revolving funds to finance their construction in advance of receiving their cash incentive, and monitor latrine use post construction. But, initiatives around funds mobilisation for poor were not observed being undertaken either by the NGOs or the VWSC. Some discrimination against SC people’s nomination to VWSC by villagers was noticed (see quotes Table 2, VWSC membership: exclusion of low caste).

A single training of one half to 1 day was convened by each NGO for 2–3 members invited per VWSC (mostly President and Secretary). A resource person from the intermediary organisation mostly imparted these trainings, which lasted for only 2–3 h. Any module nor any schedules were observed being prepared or followed for the training, indicating lack of advance planning or designing of the sessions. The attendees did not seem to take the training seriously, and often the female attendees reached late to the venue, delaying the start by 1–2 h. More effort was seen being laid on preparing lunch for the attendees than conducting the training. The learning from these trainings was to be shared with other VWSC members but in the IDIs, members frequently expressed lack of clarity on their roles and responsibilities.

#### *Kumari *committees and sanitation promotion

*Kumari* Committee as a concept was conceived by the lead agency and previously implemented in the TSC villages, which was replicated in NBA villages. It is an adolescent (unmarried) girls group formed in each village, where its members are expected to reach out to other households in the village, motivate and encourage the family members to adopt and use latrines. Without any restrictions on committee size, membership varied depending on the number of unmarried adolescent girls living in the village. The NGO leaders and it’s senior staff regarded *Kumari* Committees, to be playing an important role in generating demand for latrine, which was based on their belief that adolescent girls have a particular persuasion power on parents (see quotes Table 2, *Kumari* Committee: perceived value). In contrast to NGO leadership, CCs and GPSFs seemed far less convinced of this concept. They remarked of forming these committees upon receiving instructions from the intermediary organisation, without fully understanding the committee’s purpose and it’s member’s roles and responsibilities. No training programme was specifically organized for the members and members seemed to have not understood their roles and responsibilities.

Documentation of committee’s formation, resolutions, guidelines for membership or activities report were not found, except the research team witnessing the one time drawing competition held among the members, following instruction from the intermediary organisation. In these competitions, participating members were asked to draw the WASH situation of their village, but the members were unable to relate the idea of drawing competition with the latrine promotion, though NGO staff believed such competitions would sensitise the members to maintain a hygienic and dignified life. As indicated in quotes (see Table 2, *Kumari* Committee: lack of purpose), the committee’s activities appeared to have been implemented primarily to fulfil instructions from higher up.

#### Social mapping exercise

Social mapping (Activity 4, Fig. 1) was supposed to be held in village’s any public place engaging maximum villagers possible, in drawing a map of their village identifying habitations, streets, important landmarks and locating all open defecation sites with the aim to raise awareness on adverse impacts of open defecation. In the absence of government’s specific guideline for social mapping, variations were observed in the way the mapping exercise was carried out by the NGO field staff. Some mobilized ‘only villagers (around 50 – 60)’, while others involved ‘only VWSC members (around 6–7)’, some fully entrusted the 'VWSC' to prepare whereas, some sketched the map themselves and finalised it later in consultation with VWSC members. Some were creative in drawing the map, identifying and labelling OD areas as “contamination points”, with the aim to draw a comparative map 20 months post this sanitation intervention, to show people the changes in the village’s sanitation situation (see quotes, Table 2, Social mapping: used to track open defecation). The time spent preparing the maps varied between few hours to several days.

In one NGO, none of the staff had experience preparing social maps. Despite their requests to the intermediary organisation for trainings especially on PRA and mapping, it was not held. One CC admitted not knowing what a social map comprised of, its purpose or the preparation process, explaining he was entrusted by his organisation to draw the maps because he was good at map drawing. Many villagers revealed of not knowing about their village’s social map preparation. Some villagers participated in these mapping exercises because they felt somewhat coerced (see last quote, Table 2, Village awareness meeting: fixation with construction subsidies), but were not able to explain the purpose of these maps and correlate with sanitation.

#### Wall painting

**Fig. 2:**
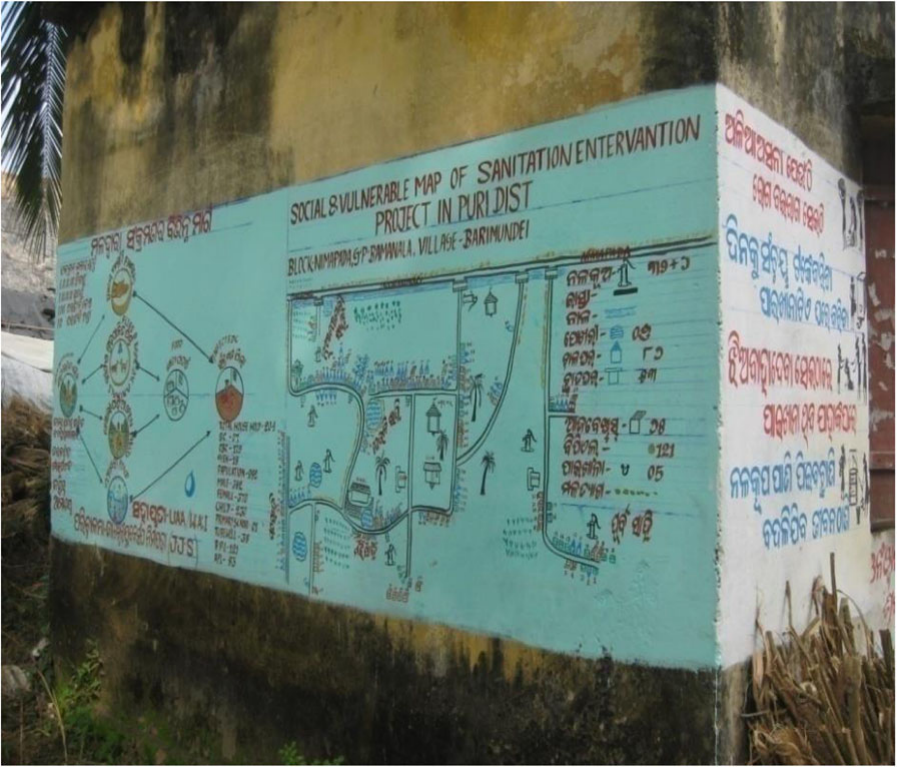
Wall painting in a study village

A wall painting was drawn as a part of mobilisation activities (see example, Fig. 2) at a strategic location in the village, facilitated by NGOs with funding support from lead agency. The painting had two elements: the F-Diagram of faecal - oral transmission pathways of diarrhoea pathogens [30], and the village’s social map. A few wall paintings included slogans on the impacts of open defecation and the importance of latrine use. These paintings were believed to open villagers’ eyes to their village’s poor sanitation situation and the negative health impacts of open defecation. However, the F-Diagram was not explained to villagers, and therefore many failed to understand or explain it to us. In some villages, these paintings created a negative impression, and rumours were spread against the NGO misusing latrine construction’s financial incentive for the wall paintings (see quotes Table 2, Wall painting: dissonant NGO and villager perceptions).

#### Micro plan

A ‘micro plan’ was prepared for each village by the NGO staff. Together with a few villagers and sometimes involving VWSC members, they collected past 20 years data on people’s lifestyle, their health and education status, drinking water provisions in the village, which was then mapped to see the development in the past years and then identified the present needs. The staff could not explain the objective behind the preparation of the micro-plan, how the data or plan would be used or integrated into other sanitation promotion activities but informed of receiving instructions from higher ups to collect data in a prescribed format (covering the above mentioned aspects). During interviews, villagers and VWSC members expressed their unawareness of any micro plan prepared for their village and had not heard the term ‘micro plan’ before.

## Discussion

This research shows the community mobilisation and IEC activities (meant to generate demand for latrines, encourage latrine adoption and eliminate open defecation behaviour) in the villages, implemented in both the versions of the sanitation campaigns - TSC and NBA, differed little [29]. Similarities were many that included the programmatic arrangements where the lead agency -WaterAid collaborated with the same intermediate organisation - UAA to execute the campaigns, and the intermediate organisation further delegating the village level sanitation promotion to the local NGOs. The implementation strategy, approaches and the management of village level activities and mobilisations by the cluster coordinators with the team of motivators were same which included the introductory meetings by NGO field staff, awareness meetings, door to door household visits, formation of VWSC and Kumari committees, training of the VWSC members on the same topics, social mapping of the village, wall paintings and messaging around health impacts, environment pollution and dignity loss of women. The additional activities held in TSC but not held in NBA were the transect walks in the village and walk through the open defecation site, wealth ranking to identify poorest households and latrine allocation to households based on their economic status, field staff training on PRA techniques, selection of ‘master masons’ and their training on latrine construction. Some of the new mobilisation activities tried in NBA were the interface meetings and micro plan preparation. The major difference were the roles and responsibilities of the field staff implementing these campaigns. In NBA staff were confined to conducting sanitation promotions and mobilisations, whereas in TSC, they had additional responsibilities of facilitating household latrine construction and procurement of construction materials [29].

The mobilisation activities were not well received by villagers in NBA implemented villages is evident, as people were mainly interested in the enhanced financial incentive for latrine construction. The infrastructure building orientation was a major weakness of the TSC which the NBA was designed to overcome, but in practice at least in this case, it has not [31, 32]. NGO staff was assigned latrine targets although household latrine construction was not their responsibility, resulting in a strong bias on pushing latrine construction in village awareness meetings. Several important logistical and organisational weaknesses observed in the approaches to promote sanitation under NBA, many of which have been noted previously in the context of TSC [11] are discussed below.

### Implementation strategy

A well defined implementation strategy was not laid out by the implementers – both the intermediary and the local NGOs for NBA’s mobilisation and sanitation promotion, which is reminiscent of TSC’s inconsistent implementation of community mobilisation and IEC activities in the first 50 villages [29] and in other parts of India [9, 11, 12]. There were neither clear government instructions or guidelines for the NGOs to follow, nor any monitoring mechanism by government to track NGO’s work and deliverables. Evaluations have found IPC to be the most effective communication method within IEC to persuade people and create demand for sanitation [12], which again proved to work well with home visits for sensitising all household members. The GoI’s ‘Sanitation and Hygiene Advocacy and Communication Strategy Framework 2012-17’ [33] emphasises IPC at the grassroots level, but our research shows, staff were not trained on IPC and facilitation skills resulting in non use of this framework.

Post mobilisation activities, presence of a supply side, sanitation marts or a supply chain strategy and some mechanism to obtain credit would have encouraged the motivated villagers to access the latrine construction materials and services or obtain credit to pre-finance their latrine construction, but it’s complete absence during this NBA implementation is a major oversight in any sanitation programme aiming to achieve gains in improved latrine coverage.

### NGO – Government collaboration

Though local NGOs were implementing NBA on behalf of the government, block level government officers were not officially introduced by the lead agency or the intermediary organisation about the NGO partners. This official recognition of NGOs and their field staff was important, as they were the liaison point between government and villagers, and responsible for facilitating and implementing NBA’s various components. Requests by NGO staff to the intermediary organisation to organise their introduction meetings with government officials had yet to be addressed 1 year into programme implementation. The lack of NGO’s official recognition compromised their acceptability by villagers, as well by the government officials. This possibly did not motivate the NGO field staff to reach out to the government officials, engage them in interface meetings or avail their resources like IEC materials. At the other end, in the absence of any monitoring mechanism by government, officials failed to gain knowledge on how the NBA was delivered and its performance in the villages, which is a clear sign that NBA, like TSC, continues to be a poorly coordinated campaign at the local government and political level [9, 11, 32].

### Political interference

Political will or supportive political leadership is considered to be essential for sanitation promotion [34, 35]. In India, political will is strong at the national level with the Prime Minister taking special interest in sanitation. There are also cases in India where sanitation targets were achieved within a few months due to the special interest shown by a local minister [36]. In contrast in this case, some political leaders interfered to block all latrine promotion activities in few villages. During general elections held across the country and in Odisha state in May 2014, political tensions led to halting of promotional activities. In the post-election phase, some new elected representatives who had recently gained power, sometimes caused problems, as one NGO facilitator noted: “*If some villagers did not vote for a particular leader, that leader did not cooperate to approve the documents needed for government’s financial incentive reimbursement.”* Some representatives were not keen to take forward the programmes initiated by their predecessors. “*Many government programmes are passed through the local representatives, so these leaders take advantage of this situation and create hindrances in processing the subsidies”*, said a villager who was also a ward member.

### Corruption

Cases of corruption have been reported in previous sanitation programmes [38]. The discrepancies in sanitation coverage rates between the Census 2011 and the Ministry of Drinking Water and Sanitation, attest to the existence of latrines in government records that are not real [37, 38]. In 4 study villages, community members reported of past fraud by some other NGOs collecting money per house to construct latrines and then absconding forever. As a result, these villagers stated that they distrusted the present NGOs and therefore largely ignored the mobilisation activities. In another study village, the ward member along with some influential people had previously raised false bills and stole the TSC’s incentives for new latrine construction, meaning households owning latrines in government records. As a result, households were disqualified from receiving government’s financial incentive. When the NGO staff tried visiting this village to convene mobilisation meetings under the NBA, the ward member and his partners threatened these staff to not enter this village for fear of being exposed; the village was then dropped from the NBA implementation.

### Financial incentive for household latrines

The latrine coverage in the 50 NBA programme villages, 9 months after the completion of mobilisation activities was only 19%, an increase of 7 percentage points compared to the baseline (as per UAA’s own report), despite the increased financial incentive. This is low compared to 63% coverage achieved within a year of TSC implementation in first 50 villages [28]. There are various possible contributing factors for this lower latrine coverage. First, there were changed roles and responsibilities of NGOs in these two campaigns in organising, procuring, and transporting materials and constructing latrines. People were used to NGOs building their household’s latrine under the TSC, whereas in NBA, NGOs were no longer building or pre-financing latrines for eligible households, so people may have lacked the initiative, knowledge, technical skills, or credit/financing to procure and build latrines themselves. Second, there was no viable supply strategy in place and rural sanitation marts not set up as in TSC, which led to lack of information among interested households about the models to build, cost of construction, availability and quality of the supplies, etc. Third, there was poor communication by the NGOs about NBA’s financial incentives reimbursement modalities, so villagers were unaware when they could claim reimbursement. The guidelines stated that a household could claim the cash incentive only upon latrine completion and met the government’s design requirements. Fourth, the GoI had long recognised the financial reimbursement to be cumbersome for both the households and officials under the TSC [12], yet improvements were not made in this aspect in the NBA, as officials and NGOs facilitating, both lacked understanding on processing of the incentive [14, 39]. Further the process got more cumbersome after institutional convergence and part reimbursement from DWSM and MGNREGS. Block officials in-charge of issuing the work orders required for a household to obtain MGNREGS reimbursement of the labour contribution for latrine construction had no knowledge about the convergence and did not cooperate with the NGOs. Thus, it is evident that sanitation promotion through community mobilisation and behaviour change activities alone, failed to deliver the desired results as envisaged by the theory of change by the lead agency and intermediary organisation.

### Capacities of NGOs and intermediary organisations

One of the key issues identified in this study, corroborating earlier findings from the TSC, is the lack of trained professionals and agencies to implement sanitation campaigns at village level [9, 11, 40]. Experiences from CLTS in Bangladesh [23], a community-managed sanitation programme in India’s Kerala state [41], evaluations of the TSC [11, 12] and PHAST approach [20], all emphasise the importance of training and developing skills of programme staff to plan and carry out community-based activities effectively. Sanitation promotion activities comprising mobilisation and awareness meetings, behaviour change triggering processes and other activities to generate demand among community members require experience and facilitation skills, which the NGO staff in this study admitted to be lacking [22, 23]. Despite years of experience in WASH programme implementation, both the intermediary organisation and the NGOs did not implement any innovative mobilisation/triggering strategies and most activities were similar to that followed in the TSC. Even the messaging was built around the same old patriarchal logic of women loosing dignity by defecating in the open, which made people to perceive that a latrine is mainly important for a ‘woman’, and ‘men’ to perceive no felt need to change their defecation behaviour. These findings suggest the need for competent and experienced NGOs that are process orientated and their approach to sanitation promotion is well planned and sustainable. Additionally, the staff’s capacities in terms of improving their facilitation skills, engaging the communities in the development process, enabling them to be more innovative and creative in designing and delivering the programme components is equally important, so also the budget allocation for the staff’s capacity building.

### Socio-cultural dynamics among communities

There are significant cultural reasons for low sanitation demand in India [42]. Studies have found people continuing their age-old habits of open defecation and their sanitation behaviours being strongly influenced/ingrained with rituals, attitudes, and mindsets which tend to contribute to lower motivation and demand for latrines and inhibit the latrine use [13, 36]. In addition, these study communities were found to be socially segregated in terms of caste, hierarchy, education, occupation, income and gender. The caste discrimination and power hierarchies were practiced between men and women, and the social norms were against women participating in open forums. In addition, not all NGO staff were experienced, skilled, trained or equipped, to face these challenges in bringing people of all castes, tackle aspects of caste discrimination, mobilise people of both genders in the same forum, handle the power hierarchies/dynamics, motivate and engage all sections to participate, identify problems, and provide solutions. Overcoming the discrimination and hierarchies may require greater social cohesion and solidarity within communities, therefore skill building of NGOs’ staff is important.

### Study limitations

This was a purely qualitative study that may have benefitted from the availability of quantitative data to study the effect of the programme on latrine adoption and use. However, it was obvious from the data that coverage with functional latrines and use of latrines was very low in study villages. It can be concluded that the campaign failed as a whole. As the campaign almost uniformly failed in the study villages, we are unable to identify which aspects of the campaign may have particularly contributed to programme failure. We did not assess whether the community mobilisation has resulted in a greater desire or motivation of households to build latrines in the longer term. A follow up survey may have helped to determine whether the increase in latrine construction and use in these villages resulted from community mobilisation or from the financial incentive. Soon after we finished the data collection in 2014, NBA was adapted and renamed SBA in October 2014 with a raised financial incentive of 12,000 rupees (179$). Therefore, a follow-up survey of the impact of the NBA community mobilisation activities on increased coverage would have been confounded by potential effects from the increased incentive amount.

Qualitative studies such as this are limited by the selection bias due to purposive sampling of participants and also by the extent to which study participants are willing to provide truthful answers. For example, villagers may have exaggerated accounts of corruption and other features of programme mismanagement in order to hide the fact that they may not have been interested in sanitation to begin with. Likewise, NGO staff may have exaggerated difficulties faced in the villages to divert attention from their potentially poor implementation performance. The study was done in a single district in Odisha and further studies are needed to determine to what extent the findings reflect issues relevant for other districts where NBA has been implemented.

## Conclusions

Similar to previous sanitation programmes in India, implementation of the NBA in this setting was not community-driven. There was disconnect between programme planning at the national level, its execution in villages, and the target population of households. Mobilising communities for sanitation promotion and behaviour change entails various constraints that are hard to overcome in communities that are divided in terms of caste, gender and local politics, and where demand for sanitation is exceptionally low. It is unclear whether under current circumstances, a better designed and implemented programme would have the potential to increase latrine coverage and use in the short term. Nevertheless, our study suggests the following recommendations for future sanitation promotion in rural India: 1) A realistic strategy needs to be in place. 2) The promotional campaign should be well designed based on the latest behaviour change theory and tailored to the target population and the situation in the villages. 3) The government needs to clarify the roles and responsibilities of its different departments and water and sanitation missions and extend official recognition to implementing NGOs. 4) The promotion and mobilisation strategy must be linked to a viable and tested supply-side strategy and provisions be made for households enabling them to obtain pre-finances for latrine construction. 5) A mechanism should be in place to monitor the NGOs’ work in the field. 6) Training of NGO staff is a very important element of whole sanitation implementation; therefore more resources should be allocated to build their capacities. 7) Communities should be given incentives (“carrots and sticks”) to overcome social disparities that currently hinder sanitation adoption in rural India, i.e., incentives should be allocated in a way to encourage demand for latrines.

## Abbreviations

BPL: Below Poverty Line
CLTS: Community Led Total Sanitation
CRSP: Central Rural Sanitation Programme
DWSM: District Water and Sanitation Mission
FGD: Focus Group Discussions
GoI: Government of India
GPSF: Gram Panchayat Sanitation Facilitators
IAPL: Identified Above Poverty Line
IDI: In-depth Interviews
IEC: Information, Education & Communication
IHHL: Individual Household Latrine
MGNREGS: Mahatma Gandhi National Rural Employment Guarantee Scheme
MOU: Memorandum of Understanding
NBA: Nirmal Bharat Abhiyan
NGO: Non Governmental Organisation
PRA: Participatory Rural Appraisal
PRI: Panchayati Raj Institution
RWSS: Rural Water Supply and Sanitation
SBA: Swachh Bharat Abhiyan
SHG: Self Help Group
TSC: Total Sanitation Campaign
UAA: United Artists' Association
VWSC: Village Water Sanitation Committee
